# Selection for fewer, water- and carbon-conservative needles in black spruce trees under warm, dry climates

**DOI:** 10.1093/aob/mcaf332

**Published:** 2025-12-26

**Authors:** Julie Messier, Sabina Henry, Christina M Caruso, Nathalie Isabel, Patrick Lenz, Benjamin Marquis, William C Parker, Isabelle Aubin

**Affiliations:** Department of Biology, University of Waterloo, Waterloo, ON, Canada N2L 3G1; Department of Biology, University of Waterloo, Waterloo, ON, Canada N2L 3G1; Department of Integrative Biology, University of Guelph, ON, Canada N1G 2W1; Natural Resources Canada, Canadian Forest Service, Laurentian Forestry Centre, Québec, QC Canada G1V 4C7; Natural Resources Canada, Canadian Forest Service, Laurentian Forestry Centre, Québec, QC Canada G1V 4C7; Natural Resources Canada, Canadian Forest Service, Great Lakes Forestry Centre, Sault Ste Marie, ON, Canada P6A 2E5; Ontario Ministry of Natural Resources, Sault Ste Marie, ON, Canada P6A 2E5; Natural Resources Canada, Canadian Forest Service, Great Lakes Forestry Centre, Sault Ste Marie, ON, Canada P6A 2E5

**Keywords:** *Picea mariana*, climate change, common gardens, functional traits, performance landscape, phenotypic integration, provenance trials, selection gradient analysis, Huber value, water use efficiency, leaf nitrogen to carbon ratio

## Abstract

**Background and Aims:**

Trees are increasingly at risk of maladaptation to their environment as climates change rapidly worldwide. Although adaptive evolution through natural selection is a key mechanism by which populations and species can persist in changing environments, we have limited information regarding the phenotypic traits under selection in warm and dry environments. We answer the following research questions: (1) What ecophysiological traits are under selection in warm, dry environments? (2) Does intrapopulation trait integration affect the response to selection in the warmer, drier site? (3) Is the plastic response of traits under selection adaptive?

**Methods:**

Using *Picea mariana* (black spruce) as a case study, we studied 425 trees representing seven provenances across three 50-year-old common garden trials established along a spatial climate gradient across eastern Canada. We measured height growth rate as a performance metric, and ten traits that reflect water use, thermoregulation, structural support and photosynthetic rate.

**Results:**

All traits were under selection in at least one site, mostly in combination with other traits. For two trait combinations, the strength of selection gradients significantly increased from the colder, wetter site to the warmer, drier site: water use efficiency (WUE) with Huber value (HV), and carbon-to-nitrogen ratio (CN) with HV. In the warmer and drier site, trait–trait correlations among these three traits were largely absent, except for CN:HV in two provenances. Overall, reaction norms suggest that the plastic response was not aligned with selection for trait pairs in warm, dry climates.

**Conclusions:**

Results suggest that adaptive evolution in response to climate change in *P. mariana* may favour phenotypes with fewer needles that are conservative for water and resource use. In the seven study provenances, intrapopulation trait integration should minimally impede adaptive evolution, but plastic responses to warmer and drier conditions may constrain the expression of optimally adapted phenotypes.

## INTRODUCTION

As climates shift under global change, the growth, productivity and long-term persistence of tree species are increasingly at risk (Allen *et al.*, 2010; Choat *et al.*, 2012). This threat is particularly urgent for boreal tree species, which are experiencing climate change more rapidly than those at lower latitudes (Peng *et al.*, 2011; Aubin *et al.*, 2018; Chagnon *et al.*, 2022). Rising drought-induced tree mortality (Allen *et al.*, 2010; Choat *et al.*, 2012) indicates that phenotypic plasticity alone has not been sufficient to keep pace with ongoing climatic shifts (Malcolm *et al.*, 2002; Iverson *et al.*, 2004; Brecka *et al.*, 2018). Moreover, projected migration rates are unlikely to match the velocity of climate change (Boisvert-Marsh *et al.*, 2022). As a result, mismatches between species’ distribution and their climatic niches are becoming increasingly pronounced (Gray and Hamann, 2013; Lapenis *et al.*, 2022).

In the face of climate change, tree species can avoid extinction via migration, adaptive plasticity or adaptive evolution (Aitken *et al.*, 2008). Mechanisms of adaptation increase the frequency of phenotypic traits that enhance fitness in a given environment. Adaptation can occur either through adaptive plasticity (where environmentally driven changes in phenotype of a given genotype improve fitness) or through adaptive evolution (where heritable traits that confer a fitness advantage increase in frequency across generations as a result of natural selection). Although both mechanisms can contribute to population persistence, research on phenotypic adaptation to climate change has predominantly focused on adaptive plasticity, and thus more studies on adaptive evolution are needed to gain a more complete understanding of phenotypic adaptation to climate change in trees (Lindner *et al.*, 2010; Royer-Tardif *et al.*, 2021). Indeed, while much research has examined adaptive plasticity in populations locally adapted to warm and dry environments (e.g. Blasini *et al.*, 2021; Challis *et al.*, 2022; Andrés-Hernández *et al.*, 2023), we lack basic knowledge on what phenotypic traits are selected for (i.e. traits that lead to high fitness, high fitness component, or high performance) under warming climates in trees (Alberto *et al.*, 2013). While research on natural selection on plant phenotypes (i.e. phenotypic selection) in short-lived herbaceous species abounds (e.g. Etterson and Shaw, 2001; Etterson, 2004; Ludwig *et al.*, 2004; Donovan *et al.*, 2007, 2009), comparatively little research has quantified phenotypic selection on traits of forest tree species (e.g. Castro, 2006; De La Mata *et al.*, 2017; Warwell and Shaw, 2018). Moreover, research on phenotypic selection in trees under warm or dry climates has largely focused on seed traits and early growth stages (e.g. Ramírez-Valiente *et al.*, 2021; Costa e Silva *et al.*, 2022, 2024). To begin addressing this gap, we assess which ecophysiological traits are under selection from warming climates in mature trees of a dominant boreal species.

A second goal of the study is to explore whether phenotypic integration will affect response to selection in black spruce. Although assessing the adaptive nature of individual traits is important, phenotypic integration (i.e. trait correlations) may also affect adaptive evolution. When two traits are highly integrated the response of one trait to selection is affected by selection on the correlated trait. This integration can constrain adaptive evolution when the direction of maximum trait covariance conflicts with the direction of selection, or enhance it when they are aligned (Björklund, 1996; Schluter, 1996). In contrast, a lack of trait covariance allows traits to evolve independently (Via and Lande, 1985), provided that sufficient trait variation is present in the populations and that traits are under genetic control. This can be beneficial for populations as their response to climate change can proceed unconstrained by trait covariation. If different black spruce populations exhibit different patterns of phenotypic integration, some may display trait correlations better aligned with the direction of selection, thereby conferring a greater potential for adaptive evolution across generations. Similarly, within generations, phenotypic plasticity can either help or hinder adaption: it can be adaptive if trait expression shifts in the direction of selection and improves performance, or maladaptive if the phenotype moves away from the direction of selection and decreases performance (Bradshaw, 1965; Pigliucci, 2001; Whitman and Agrawal, 2009). Our third goal is thus to qualitatively assess if the observed patterns of plasticity were aligned with direction of selection, and therefore locally adaptive.

Using black spruce as a model system, we study ten traits across seven provenances in three 50-year-old common gardens located along a climatic gradient in Eastern Canada, with height growth rate as a performance metric. We address three research questions. (Q1) What ecophysiological traits are under selection in warm and dry environments? (Q2) Does intrapopulation trait integration affect the response to selection at the warmer, drier site? (Q3) Is the plastic response of traits under selection adaptive? To answer Q1 and evaluate the role of climate, we take a two-step approach: first we quantify selection gradients within site, and second we quantify selection for traits or trait combinations under significant selection at the warmest, driest site (Petawawa), and test whether the strength of these gradients changes consistently across the climatic gradient (from Petawawa to Chapleau to Chibougamau). To answer Q3, we test whether plasticity is locally adaptive within environments by testing whether among-environment reaction norms were aligned with selection gradients within the warm, dry site (Caruso *et al.*, 2006).

We studied traits associated with ecophysiological functions that we expect to be under selection in warmer and drier climates (Table 1). We predict that warmer and drier climates will select for traits related to (1) conservative water use (thus indirectly to conservative carbon economics and high structural support), (2) safe sap transport and (3) efficient thermoregulation (Aubin *et al.*, 2016; Boisvert-Marsh *et al.*, 2020; Sniderhan *et al.*, 2021).

**Table 1.** Traits measured, acronyms, units and ecophysiological functions associated with high trait values. The ‘Prediction’ column gives the expected relationship of each trait with warmer and drier climates. Multiple predictions are made when the trait is associated with conflicting functions. ‘B’ denotes traits measured at the branch level. See Materials and methods section for full description of traits.

Trait name and acronym	Unit	Ecophysiological function	Prediction
Leaf dry matter content (LDMC)	g g^−1^	Carbon and water conservation Structural support (high)	↑ ↑ (indirect)
Leaf mass per area (LMA)	g cm^−2^	Carbon and water conservation	↑
Twig specific density (TSD)	g cm^−3^	Sap transport safety Structural support (high)	↑↑ (indirect)
Carbon to nitrogen ratio (CN) ^B^	g g^−1^	Carbon conservation	↑
Chlorophyll concentration (CHL)	mg m^−2^	Carbon acquisition	↓ (indirect)
Water use efficiency (WUE)^B^	‰	Water conservation	↑
Stomatal density (SD)	count cm^−1^	Carbon acquisition Water use (high) Thermoregulation (high)	↓ (indirect) ↓ ↑
Huber value (HV)^B^	mm^2^ g^−1^	Water use (high)	↑
Needle cooling (NC)	°C	Thermoregulation (high)	↑
Needle length (NL)	mm	Carbon acquisition Water conservation Thermoregulation	↓ (indirect) ↑ ↓

In water-limited environments, traits promoting conservative water use are likely to increase performance and to become targets of selection. Since resource-conservative tissues typically also achieve a long duration of returns via durable leaf construction, we expect an indirect selection on higher investment in structural support. We also expect selection for sap transport safety. Although the relationship between wood density and hydraulic transport safety is complex (Chave *et al.*, 2009; Hoffmann *et al.*, 2011; Pratt and Jacobsen, 2017), higher wood density can be associated with higher xylem cavitation resistance (Hacke *et al.*, 2001; Hoffmann *et al.*, 2011), resulting in higher performance in water-limited environments (Cochard *et al.*, 2007). Finally, we expect enhanced capacity for leaf cooling (i.e. thermoregulation) to confer higher performance and thus be a target of selection in warmer climates. Indeed, temperature controls metabolic rates and can impede photosynthetic activity above 35 °C, with the threshold varying among species (Münchinger *et al.*, 2023). Boreal spruce species have shown photosynthetic inhibition above ∼42 °C (Bigras, 2000; Münchinger *et al.*, 2023). Leaves can buffer variation in air temperature to maintain near-optimal leaf temperatures (Michaletz *et al.*, 2015).

## MATERIALS AND METHODS

### Study system

We examine selection on ecophysiological traits from warming climates in mature trees of a dominant boreal species, black spruce (*Picea mariana*). Climate change is documented to affect black spruce growth (Sniderhan *et al.*, 2021; Chagnon *et al.*, 2022). This generalist species, which is present within the entire range of the North American boreal forest, occurs in a variety of habitats and soil types. It is common in habitats with wet soils such as peat bogs and swamps but also grows well on clays, loams, well-drained mineral soils and boulder pavements (Viereck and Johnston, 1990). Black spruce generally grows in cold climates with a humid to dry subhumid moisture regime. It has low drought tolerance and is shallow-rooted (Viereck and Johnston, 1990; Niinemets and Valladares, 2006). The warmer and drier conditions brought on by climate change in much of its range are expected to lead to drought stress and growth decline in parts of its range (Girardin *et al.*, 2016; Aubin *et al.*, 2018; D’Orangeville *et al.*, 2018). Climate models forecast increases in temperature in Canada (IPCC, 2018; Swart *et al.*, 2019; Wotherspoon *et al.*, 2024), leading to decreases in water availability for equivalent precipitation due to increased evapotranspiration (Dai *et al.*, 2004; Barnett *et al.*, 2005; Cholet *et al.*, 2022). Indeed, climate moisture index (CMI), the difference between annual precipitation and potential evapotranspiration, is predicted to decrease across large sections of the boreal forest (Boucher *et al.*, 2020; Wotherspoon *et al.*, 2024). Further, warmer air temperatures result in increases in atmospheric vapour pressure deficit (Ficklin and Novick, 2017; Dai *et al.*, 2018), which increases transpiration rates and can lead to soil water limitation and decreased growth (Yuan *et al.*, 2019; López *et al.*, 2021). Warming from climate change has affected black spruce growth unevenly across its range, promoting growth in the northern boreal forest, where growth is temperature-limited, while reducing growth in southern regions, where water becomes limiting (Gamache and Payette, 2004; Beck *et al.*, 2011; Sniderhan *et al.*, 2021; Chagnon *et al.*, 2022). While black spruce's growth response to climate change is documented, the phenotypic traits that sustain growth under warmer and drier conditions remain unknown. In long-lived species such as black spruce, it is particularly important to understand selection in mature trees, because most trees will experience the bulk of their lifespan as adults. Indeed, black spruce can live for up to 2000 years (Laberge *et al.*, 2000) and peak reproduction occurs between 100 and 200 years (Viereck and Johnston, 1990). At 50 years of age, the trees in this study are in fact still at an early life stage.

### Study sites

**Fig. 1.**
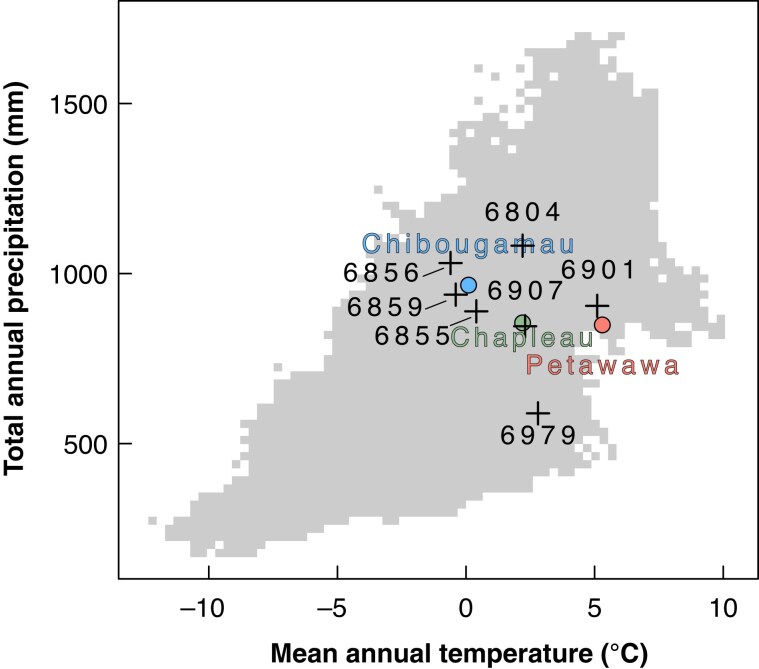
Mean annual temperature and total annual precipitation of the three provenance trial sites (pink, green, blue) and seven provenances (black crosses), averaged from 2011–40. The grey cloud represents the climate envelope of black spruce across its range in Canada. This shows that the provenances sampled here cover approximately one-sixth of the climatic space occupied by the species. Data are from McKenney *et al.* (2011) and Beaudoin *et al.* (2014).

We studied 425 trees from three sites belonging to a set of *P. mariana* provenance trials established in 1974 by the Canadian Forest Service (Morgenstern and Kokocinski, 1976; Keable, 1978). These provenance trials were established for forestry purposes to determine which of various provenances (populations of different origin from across the species’ range) grew best at various geographic locations. We selected three provenance trials that shared seven provenances and covered a climate gradient, in terms of both temperature and water availability (Supplementary Data Table S1, Fig. 1). The warmest and driest site was located in Petawawa Research Forest near Chalk River, ON. A site with an intermediate climate was located near Chapleau, and the coldest and wettest site was located near Chibougamau, QC. During establishment, each site was divided into three to six replicate blocks. Within each block, 16 trees per provenance were planted in grids of 4 × 4 trees, forming small plots (Morgenstern and Kokocinski, 1976). Since the cooperative black spruce provenance programme was designed to identify the optimal genetic stock for each site, tree spacing was adjusted among sites to maximize productivity according to local site conditions. Although the sites differ in factors other than climate, including soil properties (Table 2), height–age relationships indicate that all three of our study sites nonetheless fall within the high-productivity class characteristic of well-drained upland sites with medium to coarse, moderately fertile soils (Plonski, 1981). Soil properties were not systematically documented across sites, such that we lack comparable information across locations for a formal comparison.

**Table 2.** Site characteristics for Petawawa, Chapleau and Chibougamau. The climate moisture index (CMI) was retrieved from BioSIM (Fortin *et al.*, 2022) and averaged from 1981 to 2010. The growing season spans the months of May to September, inclusively. MAT and MAP data were retrieved from McKenney *et al.* (2011) and averaged from 1981 to 2010. Soil descriptions are from Morgenstern and Kokocinski (1976), Keable (1978) and (Morgenstern and Mullin, 1990). Productivity class is from Plonski (Plonski, 1981).

Characteristic	Petawawa	Chapleau	Chibougamau
Mean annual temperature (°C)	5.3	2.2	0.1
Total annual precipitation (mm)	849	855	966
Climate moisture index, annual and for the growing season	2.5; −0.8	3.1; 0.1	4.9; 3.1
Water-holding capacity (%)	77.7	78.1	62.4
Spacing between trees (m)	1.8 × 1.8	1.8 × 1.8	2.4 × 3
Experiment number	353-H-5	353-H-4	353-B-3
Soil description	Dry to moist, medium to fine sand, medium fertility	Dry to fresh, shallow to moderately deep, medium to fine and silty acidic sand, medium fertility	Fresh to moist, shallow, coarse to medium sand, medium fertility
Productivity class	High	High	High

The trees originated from seven provenances (Supplementary Data Table S1), selected because they were present on most sites, provided sufficient individuals for sampling at each site, and reflected the species’ phylogeographic structure linked to variation in climatic responses (Girardin *et al*., 2021). As most genetic variation in black spruce occurs within populations rather than among them (Jaramillo-Correa *et al.*, 2004; Gérardi *et al.*, 2010), these provenances capture much of the standing genetic variation, including both neutral and adaptive components. Further, they cover one-third of the species’ geographic range and one-sixth of its climatic range (Fig. 1, Supplementary Data Fig. S1). Black spruce descends from distinct glacial refugia that lead to distinct genetic lineages (Jaramillo-Correa *et al.*, 2004; Gérardi *et al.*, 2010), and the study populations are different admixtures of these lineages (Supplementary Data Table S1). We explore in our analyses whether these distinct genetic backgrounds underlie differences in trait integration among provenances. At each site, an average of 23 trees per provenance was sampled (ranging from 14 to 41). Five provenances were common to all three sites: 6804 Roddickton, NL; 6855 Matagami, QC; 6859 Parc Mistassini, QC; 6901 Bancroft, ON; and 6907 Timmins, ON. Two provenances were common to Petawawa and Chibougamau only: 6856 Manicouagan, QC and 6979 Rocky Mountain House II, AB.

### Performance metric

We used lifetime relative height growth rate of individual trees as a performance metric. Cumulative height growth provides an integrated measure of performance accumulated over five decades. In long-lived trees such as black spruce, individuals spend the vast majority of their long lifespan in the adult stage (Laberge *et al.*, 2000), such that selection occurring during this stage is highly consequential for lifetime performance. Indeed, previous work has shown that black spruce continues to respond to climate throughout its lifespan (Girardin *et al.*, 2021). By integrating performance over decades, cumulative height growth captures persistent climatic differences among sites while smoothing annual intrasite variation, thereby providing a robust basis for inferring selection along a spatial climatic gradient. Within species and environments, individual differences in growth reflect differences in resource acquisition. Consequently, growth rate has been shown to correlate with survival and reproduction, two fitness components (Farris and Lechowicz, 1990; Arntz *et al.*, 1998), and is broadly used as a performance metric in selection gradient analyses (Geber and Griffen, 2003; Caruso *et al.*, 2020) and in forestry (e.g. Younginger *et al.*, 2017; Girardin *et al.*, 2021; Robert *et al.*, 2024). Further, as Arnold (1983) emphasized, performance metrics occupy an intermediate position between morphology and fitness, such that the links between phenotype and performance are often stronger and more interpretable than those between phenotype and lifetime fitness. A meta-analysis found that neither the strength nor the direction of selection gradients on plant functional traits varied among fitness or performance components used (Caruso *et al.*, 2020). Vegetative growth rate is also the only accessible indicator of performance in mature trees in natural settings. Here, by using phenotypic selection analyses with growth rate as a performance metric, we identify the ecophysiological traits conferring a high above-ground growth rate in mature black spruce. The height of each individual tree was measured in 2022 in Petawawa, 2023 in Chapleau and 2016 in Chibougamau, and relative height growth rate since planting was calculated by dividing tree height by the age at the time of measurement (RGR; m per year).

### Sample collection

In July and August 2022 and 2023, one branch per tree was sampled from at least three blocks at each site. To standardize light exposure, in the morning of each sampling day we collected a healthy, sun-exposed, lateral branch from the top third of the tree crown using a 13.5-m telescopic pole pruner. It was then trimmed to contain the 0- to 5-year-old needles and an additional length of branch, which was placed in florist tubes filled with water for rehydration prior to trait measurements. Branches were then placed in plastic bags with a damp paper towel and stored in the dark in a cooler on ice until measurements in the afternoon (Garnier *et al.*, 2001). On average, 27 trees per provenance per site (range 15–41) were sampled, resulting in a total of 143 trees sampled at Petawawa, 156 at Chapleau and 132 at Chibougamau.

### Trait measurements

We measured ten traits associated with structural support, photosynthesis, water use and thermoregulation and known to be associated with plant response to temperature or soil moisture deficit (Table 1). Details of the relationships between each trait and its physiological function, as well as with heat or water availability gradients, are provided in the supplementary Data. Since these types of traits have a genetic basis (Geber and Griffen, 2003), we can assume that traits from needles emerging over the last 4 years reflect phenotypic variation among individuals.

Two time-sensitive traits (chlorophyll content (CHL) and needle cooling (NC)) were measured indoors in the afternoon immediately following sample collection and while the samples were still in the water-filled tubes. Branches were brought indoors and taken outside the cooler for 30 min to acclimate to ambient air temperature. Total CHL was measured on the adaxial side of needle bundles using a CCM-300 chlorophyll content meter (Gitelson *et al.*, 1999). Two measurements per needle age class were taken, except when they differed by more than 10 %, in which case a third measurement was taken. Needle cooling (NC) was determined from needle and ambient temperature measured using a TCAM-300 thermal camera, a high-accuracy temperature sensor (TMP117 High-Accuracy Digital Temperature Sensor) and FlashImagePro software (see Supplementary Data). Needle cooling values are positive when the needle is warmer than the ambient temperature and negative when it is below, with more negative NC values indicating stronger cooling.

The remaining eight traits were measured in the laboratory while branches were still in the florist tubes, within 7 d of sampling. Two perpendicular diameter measurements (mm) were taken at the base of the fourth year of growth using digital callipers. Huber value (HV) was calculated as the average branch diameter divided by the dry mass (g) of needles supported by the branch. Branches were then cut and separated into twigs based on growth year identified from terminal bud scars. For each growth year, 12–15 intact, healthy, mature needles were removed for trait measurements. Needle fresh weight was measured using analytical balances. The needle length (NL, mm) and fresh needle area (cm^2^) were then determined from scans of 600 dpi (WinSeedle V. 2020). Needle dry weights were taken after drying at 60 °C to constant weight. Leaf dry matter content (LDMC) was calculated as the ratio of dry to fresh weight of each set of needles. Leaf mass per area (LMA) for each set was calculated by dividing the dry weight by total fresh area. The total dry needle weight of each growth year was used to calculate abundance-weighted needle trait values at the branch level. To determine twig specific density (TSD), three twigs of each growth year were haphazardly sampled (excluding the largest and smallest sizes to obtain median values), and their fresh volume was determined by water displacement and then dried at 60 °C to constant weight. The TSD of each growth year was then calculated as twig dry mass (g) divided by twig fresh volume (cm^3^). Finally, branch-level TSD was determined by abundance-weighting each year’s TSD by their relative dry weight.

To measure water use efficiency (WUE), 12 needles for each age class used for previous trait determinations were ground into a fine powder using a ball mill. Stable carbon isotope analysis was performed at the Environmental Isotope Laboratory at the University of Waterloo using a 4010 Elemental Analyzer coupled to a continuous flow isotope ratio mass spectrometer. To determine the variation within individual ground samples, duplicate measurements were performed for 34 samples, spaced at regular intervals throughout measurement. Images of the abaxial surface of three needles were taken using a stereo microscope. The number of stomata was counted along the maximum visible needle length using WinSeedle software, and stomatal density (SD) was calculated as the number of stomata per unit length.

### Statistical analyses

#### Data imputation and description of trait correlations

All statistical analyses were conducted in R version 4.4.0 (R Core Team, 2025). Two per cent of all data was missing due to lost samples or measurement errors (3 % in Petawawa, <1 % in Chapleau, 3 % in Chibougamau). Since the models quantifying selection gradients fit many parameters and the package *lme()* omits any rows containing missing values, it was important to retain as much statistical power as possible and avoid losing samples due to missing data. Missing values were imputed for each site from other traits from linear mixed effect models, using the *lme()* function from the *nlme* package (Pinheiro *et al.*, 2025). The model with the highest predictive power for missing values was identified using the *dredge()* function from the *MuMIn* package (Bartoń, 2025). We took a conservative approach, only imputing missing data when the *R*^2^ of the predictive model was >60 %. Given this precaution and the fact that we imputed such a small fraction of the dataset, imputation is unlikely to have had any meaningful effects. Otherwise, the missing values were left as ‘NA' (i.e. not available). After imputation, <0.01 % of the data was missing from each site, resulting in *n* = 142 trees in Petawawa, *n* = 151 trees in Chapleau and *n* = 132 trees in Chibougamau. Since patterns of trait covariation have implications for potential model collinearity and inform the interpretation of multivariate selection, we characterized the trait correlation structure. Correlations among traits were described using *rcorr()* from the *Hmisc* package (Harrell, 2025) and principal component analyses (PCAs) using *PCA()* of the *FactoMineR* package (Lê *et al.*, 2008). PCAs were conducted on trait data across all sites as well as within each site. The statistical significance of the principal component axes was tested using *PCAsignificance()* from the *BiodiversityR* package (Kindt and Coe, 2005), which compares the amount of variance explained by each axis with the variance explained in broken-stick null models.

#### Quantifying selection gradients

To determine which ecophysiological traits are under selection in warm and dry environments, we first quantified selection gradients in each site. To assess selection at each site, we used the Lande–Arnold regression approach to measure directional (*β*) and correlational (*γ*) selection gradients at each site (Lande and Arnold, 1983), where directional gradients quantify the effect of individual traits on performance and correlational selection quantifies the effect of combinations of traits. Here, the partial regression coefficients in multiple linear regressions of height RGR against traits represent the strength and direction of selection. We standardized trait values within each site to a mean of zero and a variance of 1 (*z*-transformation) and relativized RGR values within each site by dividing individual RGR values by the mean RGR for that site (Conner and Hartl, 2004). Within each site we assessed *β*s from multiple regressions of RGRs on all ten traits with provenance as a random effect, and we assessed *γ*s from multiple regressions of RGRs on all ten traits, their two-way interactions and provenance as a random effect (Lande and Arnold, 1983). We used the *lme()* function from the *nlme* package (Pinheiro *et al.*, 2025). We confirmed the absence of multicollinearity by calculating the variance inflation factor (VIF) of the fixed terms in the mixed model, using *vif()* for the *car* package. All VIFs were below 2.3, which is below the conservative threshold of 3 (Zuur *et al.*, 2010). Following Zuur and Ieno (2016), we verified that the data met model assumptions by examining diagnostic plots.

#### Testing for climate-consistent selection gradients

Second, since we are interested in selection from warm and dry environments, we identified which gradients are significant in Petawawa, and then tested whether those selection gradients vary directionally across sites along the climatic gradient (Wade and Kalisz, 1990). We identified such patterns using two criteria: (1) the selection gradient showed clinal variation (i.e. continuous gradual change with the ecological gradient) with climate among sites; and (2) the selection gradients differed significantly between Petawawa and Chibougamau, the two sites at opposite ends of the climate gradient. As we cannot infer causation unequivocally from this observational design, we interpret only those selection gradients that change directionally across the climatic gradient, and refer to them as ‘climate-consistent’ selection gradients. We used the *tsum.test()* function from the *BSDA* package to perform two-sample *t*-tests from the coefficient estimates and their standard errors (Arnholt and Evans, 2023). While specific traits may be under selection from many factors at a given site, we interpret the clinal changes among sites as primarily driven by differences in climate. Indeed, other than differences in tree spacing, which we do not expect to impact the results (see Discussion section), we are not aware of any clinal differences in the environment among those sites.

#### Assessing phenotypic integration and its potential effects on response to selection

To evaluate whether trait integration might affect the response of the provenances to selection, we examined whether trait integration was aligned with the direction of selection. This approach makes the common simplifying assumption that the phenotypic variance–covariance matrix is a proxy for the genetic variance–covariance matrix (Merilä and Björklund, 2004). Since we are interested in selection under warm, dry climates, we used selection gradients at Petawawa, our warmest, driest site, to create performance surfaces using the *geom_contour()* function in the *ggplot2* package (Wickham, 2016). Smooth contour lines were generated using the *interp()* function from the *akima* package (Akima and Gebhardt, 2022). For each provenance, the residuals of the traits from the regression models were extracted using the *residuals()* function from the *stats* package, and correlations between the residuals were tested with the *cor.test()* function from the *stats* package.

#### Assessing the adaptive nature of trait plasticity

The small number of provenances (*n* = 7) precluded a formal test of the global adaptive nature of plasticity among environments (Van Kleunen and Fischer, 2005). Instead, we took a common approach to evaluate whether patterns of plasticity were adaptive within hot and dry environments (Caruso *et al.*, 2006): if the direction of trait shifts across sites was consistent with the direction of selection in Petawawa (the warmer, drier site), we inferred that plasticity was locally adaptive. Thus, for each trait under selection in Petawawa, we first tested for significant plastic changes in traits across sites: we performed a two-way ANOVA with site and provenance as fixed factors using *aov()* from the *stats* package, then performed a *post hoc* LSD test on site alone using *LSD.test()* from the *agricolae* package (de Mendiburu, 2023). Second, we qualitatively assessed whether the direction of plastic change along the climatic gradient was aligned with the direction of selection in the warm, dry site.

## RESULTS

### Traits under selection at each site

**Fig. 2.**
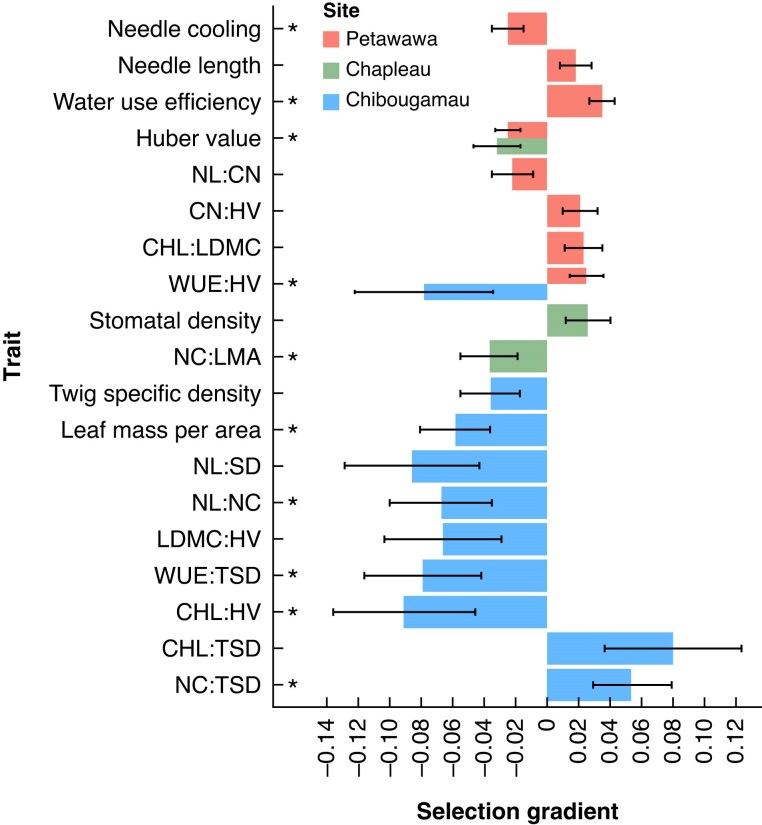
Significant and marginally significant selection gradients (*β* and *γ*) for each of the three sites. Black horizontal lines represent the standard error. Asterisks indicate significance at *P* ≤ 0.05 level. All other traits are marginally significant (0.05 < *P* ≤ 0.10). See Table 1 for explanation of abbreviations in trait combinations.

All ten traits measured were under directional or correlational selection in at least one site, for a total of 21 significant or marginally significant selection gradients across the three sites (8 in Petawawa, 3 in Chapleau and 10 in Chibougamau; Fig. 2, Supplementary Data Table S2). Of the 21 selection gradients, 7 were for individual trait values (directional selection, *β*) and 14 were for trait combinations (correlational selection, *γ*). The traits and trait combinations under selection largely differed among sites. Specifically, in Petawawa three traits were under directional selection (increased NC, increased NL and increased WUE) and five trait combinations were under correlational selection (CHL and LDMC; NL and carbon to nitrogen ratio (CN); LDMC and WUE; WUE and HV; and CN and HV).

### Climate-consistent shifts in selection on traits

**Fig. 3.**
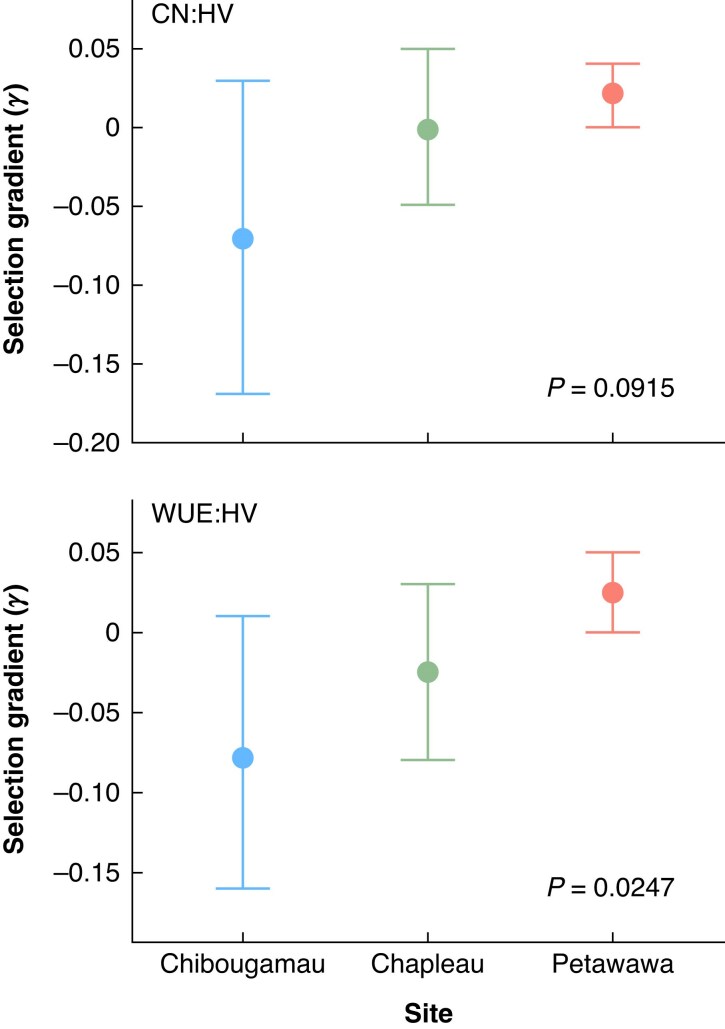
Selection gradients for trait combinations CN:HV and WUE:HV were clinal across sites and statistically or marginally different between Petawawa and Chibougamau. Bars represent the 95 % confidence intervals of each site.

The shifts among sites were consistent with a role for climate in two of the eight selection gradients in Petawawa: trait combinations CN:HV and WUE:HV. Selection for the CN:HV trait combination showed a marginally significant clinal increase from non-significant in Chibougamau to +0.021 in Petawawa (*P* = 0.092). Selection for the WUE:HV trait combination showed a significant clinal increase from −0.078 in Chibougamau to +0.025 in Petawawa (*P* = 0.025) (Fig. 3, Supplementary Data Table S2). Changes in the other selection gradients in Petawawa were either not clinal or not significantly different from Chibougamau (Supplementary Data Fig. S3).

### Phenotypic integration within provenances

Intraspecific integration in black spruce was weak in the set of provenances sampled, with the strongest correlations found between LDMC and LMA (*r* = 0.42, *P* < 0.0001, Supplementary Data Table S3). In both the experiment-wide and within-site PCAs, principal component axes were not significant, and the first two axes accounted for a small fraction of the total variation (all data, 34 %; Petawawa, 3 %; Chapleau, 38 %; Chibougamau, 42 %; Supplementary Data Fig. S2). The site-specific PCAs for Chibougamau were similar to the experiment-wide PCA, but the Petawawa and Chapleau PCAs differed from each other and from the experiment-wide PCA (Supplementary Data Fig. S2B).

**Fig. 4.**
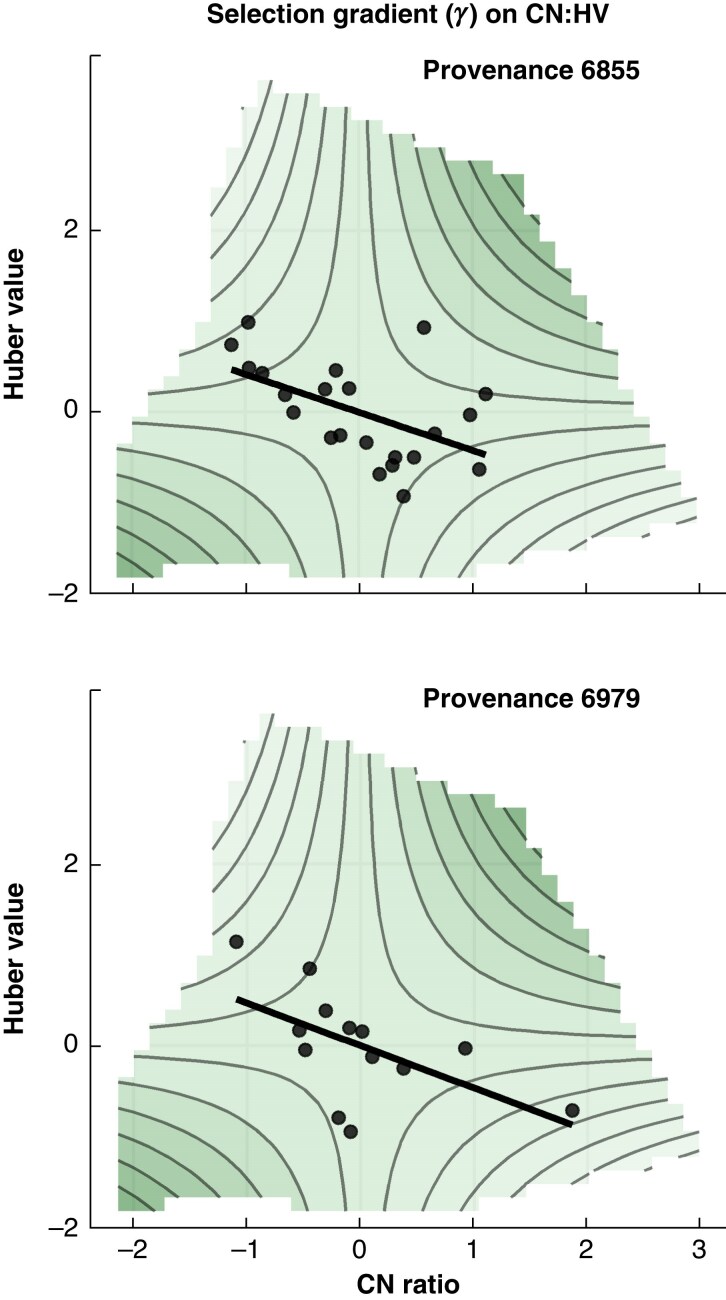
Significant intraprovenance trait correlations for trait combination CN:HV in provenances 6855 (Matagami, QC) and 6979 (Rocky Mountain House II, AB).

Since results indicated climate-consistent selection gradients for CN:HV and WUE:HV combinations, we explored the intraspecific trait correlations within provenances for those trait pairs aligned with the direction of selection by examining correlations among the residuals of WUE, HV and CN. The trait residuals were not correlated within provenances except for significant negative correlations between HV and CN in provenances 6855 (*P* = 0.012) and 6979 (*P* = 0.042). The negative correlation between HV and CN in both provenances is largely perpendicular to the direction of selection, which favours combinations of high values of both traits (Fig. 4).

### Plastic responses across sites

**Fig. 5.**
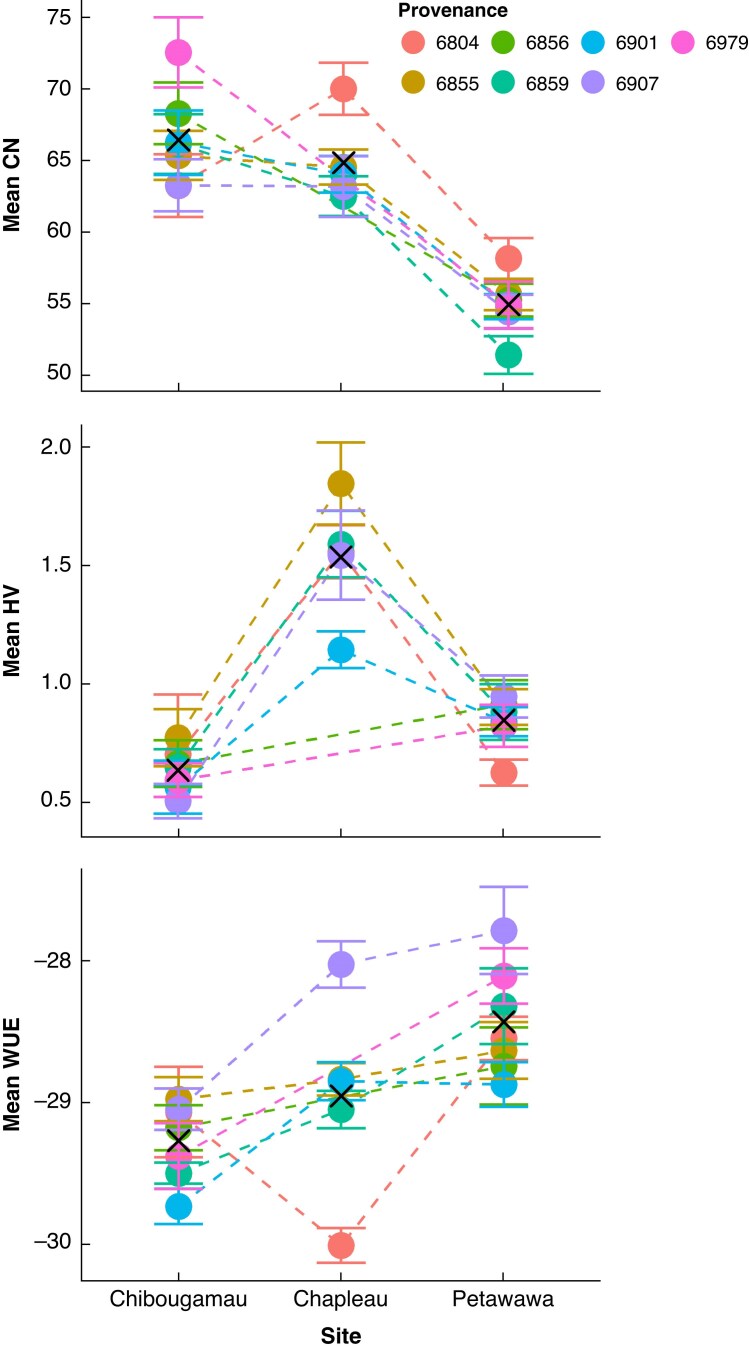
Comparative reaction norms of provenances across sites for the three traits under climate-consistent selection. Black crosses show site means. For a given trait, sites with different letters indicate significantly different means (*P* < 0.05). See Supplementary Data Table S1 for provenance information.

All traits showed a significant plastic response across sites, with most provenances showing similar patterns of trait variation among sites (Fig. 5, Supplementary Data Fig. S4). Since results indicated climate-consistent selection for positive interaction among CN:HV and WUE:HV, plastic increases in CN, HV and WUE would suggest that plasticity improves performance within hot and dry environments. Results show that WUE increased plastically from the coldest to the warmest site, following the direction of selection (*P* ≤ 0.0001). In contrast, CN ratio decreased from the coldest to the warmest site (*P* ≤ 0.0001). Huber value showed a non-linear plastic response across the climate gradient, with highest values at Chapleau (the site with intermediate climate, *P* ≤ 0.0001, Fig. 5). Thus, the plastic response of CN ratio and HV to the spatial gradient does not track the direction of selection at the warmer, drier site.

## DISCUSSION

Using *P. mariana* (black spruce) as a case study, we combined classical selection gradient analyses and provenance trials established along a spatial climate gradient to identify the ecophysiological traits that enhance individual growth performance in warm and dry environments, and to evaluate the extent to which phenotypic integration and plasticity may facilitate or constrain adaptive responses. Since adaptive evolution is one of three mechanisms by which species persist under climate change, understanding what is selected for and whether phenotypic integration and plasticity are expected to hinder or facilitate that response is important to anticipate and mitigate the impacts of climate change on tree species. We found that individual performance depends on trait combinations and that the traits under selection differed among sites, indicating that natural selection varies across environments. This indicates that understanding trait–performance relationships requires measuring multiple traits and accounting for environmental heterogeneity. Two trait combinations showed climate-consistent selection, suggesting that fewer needles with conservative water and resource use are favoured in warmer and drier climates. These traits are consistent with a response to water limitation over a response to heat as a selective agent. Within the seven provenances studied, phenotypic integration was weak, suggesting limited constraint on adaptive evolutionary responses for these provenances. In contrast, plasticity in these traits did not consistently align with the direction of selection in Petawawa, suggesting that plastic responses may not maintain growth performance under warm and dry climates. These results are consistent with other studies finding poor acclimation to warming in conifers (Way and Yamori, 2014).

### Individual-level performance depends on trait combinations and environmental context

We found that all ten ecophysiological traits studied here were under selection in one of the three sites, whether individually or in combination with another trait. This finding has several implications for trait-based plant ecology. First, although functional traits are defined as individual-level traits that affect performance and fitness components (Violle *et al.*, 2007), the adaptive nature of many commonly measured ‘functional’ traits often remains an untested assumption, with recurrent calls for its validation (Ackerly *et al.*, 2000; Ackerly and Monson, 2003; Shipley *et al.*, 2016; Salguero-Gómez *et al.*, 2018; Swenson *et al.*, 2020). Our findings that the ten study traits were associated with growth performance of black spruce in at least one site thus build on emerging evidence that commonly measured functional traits indeed affect performance and fitness components (Geber and Griffen, 2003; Caruso *et al.*, 2020). Second, most selection gradients were for trait combinations, which indicates that a given trait value is only adaptive when it occurs in combination with another trait value. A corollary is that measuring many traits was necessary to detect selection. These findings thus suggest that assessing the individual effects of few traits could be one reason why many studies fail to find relationships between traits and individual performance or demographic rates. For example, studies examining the effects of three to five traits on individual growth rates (Adler *et al.*, 2014; Paine *et al.*, 2015) and population demographics (García Criado *et al.*, 2023) found no or weak relationships. Third, our finding that traits under selection differed across sites indicates that natural selection differs among environments. This supports previous conclusions that the environment needs to be considered at a sufficiently detailed spatial scale to properly detect relationships between traits and performance (Shipley *et al.*, 2016; Swenson *et al.*, 2020). Indeed, studies conducted across broad environmental gradients and linking traits to performance at the individual level may find weak relationships because environmental heterogeneity obscures them (e.g. Paine *et al.*, 2015). Lastly, the fact that only some traits associated with a given function were under selection at a site suggests that selection may act on a specific aspect of that physiological function. This implies that detecting selection on a specific function in a species may require measuring multiple traits that represent different aspects of that function. For example, in Petawawa we detected selection for high WUE, but not for other traits directly affecting water use (such as SD) or drought tolerance (such as LMA). This is unfortunate, as structural traits such as LMA are much faster and less expensive to measure than intrinsic WUE estimated by stable carbon isotope composition. Characterizing the ecological strategies of plants by measuring a few common traits is a goal of trait-based ecology (Westoby *et al.*, 2002; Pérez-Harguindeguy *et al.*, 2013).

Taken together, these results highlight a central implication for trait-based ecology: the need for a detailed phenotypic assessment in this intraspecific-scale study suggests that meeting this goal may be limited to studies with broad phylogenetic and spatial scopes, as traits may only become correlated into trait axes when they span a large range of values (Funk and Cornwell, 2013). As trait axes commonly identified in broad, multispecies datasets may not hold within species (Messier *et al.*, 2017, 2018; Anderegg *et al.*, 2018), this underscores that within-species analyses may require more comprehensive phenotypic assessment than is typical in multispecies, trait-axis-based frameworks.

### Two trait combinations showed climate-consistent selection

The strength of selection for two trait combinations, CN:HV and CN:WUE, increased in warmer and drier sites (Petawawa; Fig. 3). The trees growing best in Petawawa thus had a combination of high WUE, HV and CN. To the degree that these traits are heritable, and that total height growth is correlated with fitness, our results suggest that black spruce populations may evolve these trait combinations in response to selection pressures exerted by warming and drying climates. High intrinsic WUE indicates a conservative water use, with many tree species showing higher WUE in drought-adapted populations (Hajek *et al.*, 2016; Rosas *et al.*, 2019; Ahrens *et al.*, 2020; Csilléry *et al.*, 2020; Anderegg *et al.*, 2021; Rabarijaona *et al.*, 2022; Lochin *et al.*, 2024), or plastic increases in WUE in response to drought conditions (Craven *et al.*, 2013; Limousin *et al.*, 2015; Forner *et al.*, 2018; Schimpl *et al.*, 2019). A high HV indicates a superior hydraulic supply capacity per leaf area. In our study, high HV resulted mainly from the presence of fewer needles because variation in HV in Petawawa was more strongly correlated with total needle biomass (*r* = −0.79, *P* ≤ 0.0001) than branch diameter (*r* = −0.49, *P* ≤ 0.0001) and total needle biomass was more variable than branch diameter (CV = 71 and 29 %, respectively). Lower needle biomass could result either from lower production or from loss of needles during drought events. High HVs have been found to be associated with drought conditions in many other species (Mencuccini and Grace, 1995; Li *et al.*, 2019; Rosas *et al.*, 2019; Mencuccini *et al.*, 2019; Anderegg *et al.*, 2021). Fewer needles may also be beneficial under water limitation by decreasing the need for transpirational cooling as a means of thermoregulation. High CN reflects relatively higher investment in structural support than metabolic processes. Collectively, these findings indicate that fewer water- and resource-use conservative needles is a beneficial strategy for black spruce in warm, dry environments. In this experiment, Petawawa was the warmest and driest site, such that we cannot separate adaptation to heat from adaptation to drought. Nonetheless, the three traits under climate-consistent selection are more consistent with adaptation to drought, which is consistent with literature showing that black spruce is water-limited in the southern margin of its range (Sniderhan *et al.*, 2021; Chagnon *et al.*, 2022).

Some caveats are inherent to the experimental design. First, the initial tree spacing was higher in Chibougamau than the other two sites and this factor covaries with the climate gradient. The wider spacing likely decreased resource competition among individuals and decreased mortality. Indeed, when the provenance trials were established, tree spacing was adjusted at each site to maximize tree growth (Morgenstern and Kokocinski, 1976). Differences in spacing are unlikely to have strongly affected the traits measured here because we sampled light-exposed needles in all sites. Further, the traits showing significant clinal shift in selection across sites define trees with few needles and with conservative water and resource use. This is consistent with water and heat stress, but not with limited light availability. Second, edaphic properties also differ among sites. This is an inherent limitation of observational studies, particularly legacy trials, because they were not designed with the current use in mind. Since edaphic properties can affect both the phenotype and growth rate, these differences likely added noise to the relationships between trait values and performance. Third, our performance metric, lifetime height growth rate, does not take into consideration below-ground growth, which is not necessarily proportional to above-ground growth. This may also weaken the trait–performance relationships. Overall, these limitations are expected to add noise rather than produce spurious patterns. Further, as additional noise produces more conservative estimates of climate-consistent selection, our study is thus likely to have underestimated trait selection from warm and dry climates. Nonetheless, we refer to the selection gradients as climate-consistent gradients to reflect that our experimental design allows inference of consistency with climate but cannot ascribe causation.

### Weak phenotypic integration indicates limited constraints on adaptive responses in our study provenances

Our results suggest that response to selection for the seven *P. mariana* provenances under study is largely unconstrained by phenotypic integration. Not only is the overall strength of trait integration weak, as shown by the trait correlation matrix (Supplementary Data Table S3) and the PCAs within sites (Supplementary Data Fig. S2), but only two of the seven provenances showed significant intraprovenance trait correlations for one of the two trait combinations under climate-consistent selection (Fig. 4). The two trait correlations were largely perpendicular to the direction of selection, indicating that phenotypic integration would hinder evolutionary response to selection. However, given the rarity of these instances overall (only 2 out of 21), phenotypic integration is unlikely to meaningfully affect the response to selection of these provenances of *P. mariana* under warmer climates. Over evolutionary time, given unknown future selective forces, low integration can be beneficial as it provides more flexibility for a species’ phenotype to respond to selection. A corollary to the weak trait integration in all provenances is that their integration did not differ. Thus, except for the Matagami (6855) and Rocky Mountain House II (6979) provenances, which are expected to evolve more slowly than the five others do in response to selection for high CHL:HV, none of the studied provenances showed a particularly high potential for adaptive evolution in the face of warming climates. If these results hold more broadly, this would be consistent with Robert *et al.* (2024), who report that the productivity of all populations will likely decrease by the end of the century. Provenance 6979 contains the largest proportion of western genetic lineage, which has been found to contribute to reduced population productivity in some common gardens (Girardin *et al.*, 2021). In contrast, the other population exhibiting a pattern of trait integration (provenance 6855) is one of five with a high proportion of the central genetic lineage. Thus, there is no clear signal of climate refugia lineages in the intrapopulation integration of CHL and HV. Given the absence of correlations for most traits under selection in most of the populations, our findings suggest that trait integration is not a main factor affecting response to climate change in these provenances. Although the provenances sampled here span only part of the geographic and climatic range of black spruce, most genetic variation in this species occurs within populations rather than among them (Jaramillo-Correa *et al*., 2004; Gérardi *et al*., 2010), suggesting that these seven provenances capture much of the standing genetic variation relevant to adaptive responses. Nonetheless, extending these analyses to provenances from additional climatic and geographic regions is necessary to robustly test the generality of these findings.

### Plasticity was not consistently aligned with the direction of selection

Although all traits exhibited plastic responses across sites, only WUE appears to change in the direction favoured by selection at the warmer, drier site, whereas CN and HV did not show a directional plastic response along the climatic gradient aligned with selection. Similar to other studies, we found mixed results, with evidence suggestive of both adaptive and maladaptive components (Caruso *et al.*, 2006; Ramírez-Valiente *et al.*, 2021). The plastic increase in WUE across the spatial climate gradient may indicate an adaptive response in that trait, consistent with temporal increases in WUE documented in the water-limited southern part of the black spruce range (Sniderhan *et al.*, 2021), and with patterns reported in other species (Craven *et al.*, 2013; Limousin *et al.*, 2015; Forner *et al.*, 2018; Schimpl *et al.*, 2019). In contrast, the plastic decrease of CN in warmer sites runs counter to the direction of selection, which could suggest a maladaptive plastic response of CN to warming. However, since CN is also affected by the amount of available nitrogen (Lambers *et al.*, 2008), trait differences among sites might also be driven by soil differences, which would mask the plastic response of CN to climate. Huber value showed a non-linear response, with highest values at the intermediate site. The reasons for this pattern remain unclear, since no disturbance has been documented at Chapleau recently. Leaf shedding in response to drought has been documented in conifers (Kouki and Hokkanen, 1992; Żytkowiak *et al.*, 2005; Nadal-Sala *et al.*, 2021), but the severity of the water limitation necessary to trigger this mechanism is unclear. Alternatively, trees growing under warmer and drier climates may simply produce branches with less foliage. Overall, since we found selection to act on trait combinations, not on traits alone, adaptive plasticity would require both traits in each selected pair to shift in the direction favoured by selection. This was not observed for either trait pair, suggesting that the plastic responses observed were not consistent with within-environment adaptive plasticity.

### Future work

Understanding how adaptive evolution shapes the response of long-lived tree species to warming climates remains a major knowledge gap, and our findings provide a foundation that future work can build upon across broader provenances, traits and environmental contexts. Since genetic variation within provenances is greater than among provenances, the limited intrapopulation trait integration we observed is unlikely to be specific to the seven populations studied, but broader geographic and climatic sampling is required to confirm whether this pattern is general for black spruce. In parallel, future work using complementary performance metrics or a broader suite of traits could identify other targets of selection beyond those detectable with above-ground traits and height growth alone. Promising directions include assessing selection on root traits, which increasingly appear to mediate responses to warmer and drier climates (Laughlin and Messier, 2015; Messier *et al.*, 2024), examining selection in mixed forest communities where species interactions influence performance (Alberto *et al.*, 2013), and disentangling the effects of high temperature versus water deficit, given that some populations experience strong water limitation while others do not (D’Orangeville *et al.*, 2018; Sniderhan *et al.*, 2021; Chagnon *et al.*, 2022).

Understanding how long-lived species will respond to rapid climatic change requires studying not only migration and adaptive plasticity, but also adaptive evolution, and doing so at the adult life stage where most individuals spend the majority of their lifespan. In this paper, we show how our approach, combining provenance trials with phenotypic selection gradient analyses, provides a powerful framework for detecting which trait combinations enhance performance in warmer and drier climates, and for evaluating the potential roles of adaptive evolution, phenotypic integration and plasticity in shaping future population trajectories. Provenance trials provide a more accurate assessment of phenotypic selection from climate in natural settings than short-term studies performed under greenhouse conditions (Poorter *et al*., 2016). Selection gradient analysis has rarely been used to assess phenotypic traits under selection from climate change in trees (Warwell and Shaw, 2018, 2019; Ramírez-Valiente *et al*., 2021), and to our knowledge this is the first study to do so for ecophysiological traits in mature trees in natural settings along a climate gradient. Since controlled experiments on adult trees are limited and generation times are long, existing provenance trials offer considerable potential for advancing our understanding of adaptive responses in ecologically and commercially important tree species.

## Supplementary Material

mcaf332_Supplementary_Data

## Data Availability

The trait and height growth rate data used in this study are openly available at https://doi.org/10.5281/zenodo.15571506.

## SUPPLEMENTARY DATA

Supplementary data are available at *Annals of Botany* online and consist of the following. Table S1: characteristics of the seven study provenances. Table S2: traits and trait combinations under selection across the three study sites. Table S3: Pearson correlation coefficients among standardized study traits calculated across all sites. Figure S1: geographic location of the three provenance trials and the seven study provenances. Figure S2: PCA of trait values across sites and within each site. Figure S3: selection gradients across sites for the trait and trait combinations under significant and marginally significant selection at Petawawa. Figure S4: comparative reaction norms of provenances across sites for all the study traits. Supplementary Methods: trait measurements.
