# Supplementary material for: Selection for fewer, water- and carbon-conservative needles in black spruce trees under warm, dry climates
Source: Ann Bot. 2025 Dec 26;138(2):400–14. doi: 10.1093/aob/mcaf332 (PMC13454728; doi:10.1093/aob/mcaf332)
Supplement: Supplementary file 1 [file mcaf332_supplementary_data.docx]

# SUPPLEMENTAL INFORMATION FOR:

# Selection for fewer, water- and carbon-conservative needles in black spruce trees under warm, dry climates

Julie Messier^†*^, Sabina Henry^†^, Christina M. Caruso, Nathalie Isabel, Patrick Lenz, Benjamin Marquis, William C. Parker and Isabelle Aubin

^†^ co-first authors. Equal contribution

*Corresponding author email address: julie.messier@gmail.com

## Supplementary Tables and Figures

Table S1. Characteristics of the seven study provenances. Latitude and Longitude are reported in decimal degrees. Elevation are reported in m above sea level. Mean Annual temperature (MAT) are reported in degrees Celsius. MAT: Mean Annual Temperature (° C). TAP: Total annual precipitation (mm). CMI: community moisture index reported annually and for the growing season (May to September). MAT and MAP data retrieved from McKenney *et al.* (2011), and averaged from 1981-2010. The W, C and E give the fraction of the provenance’s genotype from the Western, Central and Eastern climate refugia lineages, respectively (Girardin *et al.* 2021).

| **No.** | **Location** | **Long.** | **Lat.** | **Elev.** | **MAT** | **TAP** | **CMI** | **W** | **C** | **E** |
| --- | --- | --- | --- | --- | --- | --- | --- | --- | --- | --- |
| 6804 | Roddickton, NL | 50.90 | -56.10 | 51 | 2.2 | 1082 | 6.8; 3.8 | 0.01 | 0.24 | 0.75 |
| 6855 | Matagami, QC | 49.62 | -77.75 | 267 | 0.4 | 889 | 3.9; 2.0 | 0.02 | 0.70 | 0.29 |
| 6856 | Manicouagan, QC | 50.67 | -68.77 | 437 | -0.6 | 1031 | 4.6; 2.4 | 0.02 | 0.71 | 0.28 |
| 6859 | Parc Mistassini, QC | 50.45 | -73.63 | 400 | -0.4 | 938 | 4.7; 3.0 | 0.01 | 0.70 | 0.28 |
| 6901 | Bancroft, ON | 45.17 | -77.17 | 279 | 5.1 | 905 | 3.1; -0.7 | 0.02 | 0.73 | 0.25 |
| 6907 | Timmins, ON | 48.53 | -81.42 | 277 | 2.3 | 845 | 3.1; -0.1 | 0.01 | 0.88 | 0.11 |
| 6979 | Rocky Mtn. House II, AB | 52.37 | -115.25 | 1125 | 2.8 | 589 | 1.2; 0.9 | 0.82 | 0.12 | 0.06 |

Table S2. Traits and trait combinations significantly (p ≤ 0.05) and marginally significantly (0.05 ≤ p ≤ 0.10) under selection across the three study sites. CI = confidence intervals.

| **Trait** | **β or γ** | **p- value** | **CI** | **Std. Error** | **Site** |
| --- | --- | --- | --- | --- | --- |
| Needle length | 0.018 | 0.07 | 0, 0.04 | 0.01 | Petawawa |
| Needle cooling | -0.025 | 0.01 | -0.04, -0.01 | 0.01 | Petawawa |
| Water use efficiency | 0.035 | 3.1E-5 | 0.02, 0.05 | 0.008 | Petawawa |
| Huber value | -0.025 | 3.6E-3 | -0.04, -0.01 | 0.008 | Petawawa |
| CHL:LDMC | 0.023 | 0.06 | 0, 0.05 | 0.012 | Petawawa |
| NL:CN | -0.022 | 0.10 | -0.05, 0 | 0.013 | Petawawa |
| WUE:HV | 0.025 | 0.03 | 0, 0.05 | 0.011 | Petawawa |
| CN:HV | 0.021 | 0.06 | 0, 0.04 | 0.011 | Petawawa |
| Huber value | -0.032 | 0.03 | -0.06, 0 | 0.015 | Chapleau |
| Stomatal density | 0.026 | 0.06 | 0, 0.05 | 0.014 | Chapleau |
| NC:LMA | -0.037 | 0.04 | -0.07, 0 | 0.018 | Chapleau |
| LMA:HV | 0.04 | 0.10 | -0.01, 0.09 | 0.024 | Chapleau |
| Leaf mass per area | -0.058 | 0.01 | -0.1, -0.02 | 0.022 | Chibougamau |
| Twig specific density | -0.036 | 0.07 | -0.07, 0 | 0.019 | Chibougamau |
| CHL:HV | -0.091 | 0.04 | -0.18, 0 | 0.045 | Chibougamau |
| CHL:TSD | 0.080 | 0.07 | -0.01, 0.16 | 0.043 | Chibougamau |
| NL:NC | -0.067 | 0.04 | -0.13, 0 | 0.032 | Chibougamau |
| NL:SD | -0.086 | 0.05 | -0.17, 0 | 0.043 | Chibougamau |
| NC:TSD | 0.054 | 0.03 | 0.01, 0.1 | 0.025 | Chibougamau |
| LDMC:HV | -0.066 | 0.08 | -0.14, 0.01 | 0.037 | Chibougamau |
| WUE:HV | -0.078 | 0.08 | -0.16, 0.01 | 0.044 | Chibougamau |
| WUE:TSD | -0.079 | 0.04 | -0.15, -0.01 | 0.037 | Chibougamau |

Table S3. Pearson correlation coefficients (r) among standardized study traits calculated across all sites, color-coded to the strength or the correlation coefficient to highlight the strongest relationships (red = negative, blue = positive).

|  | CHL | NL | NC | LDMC | LMA | WUE | CN | HV | SD | TSD |
| --- | --- | --- | --- | --- | --- | --- | --- | --- | --- | --- |
| Chlorophyll concentration (CHL) | 1.00 | 0.29 | -0.07 | -0.13 | -0.12 | -0.11 | -0.36 | -0.12 | -0.09 | -0.06 |
| Needle Length (NL) | 0.29 | 1.00 | 0.02 | -0.10 | 0.01 | -0.05 | -0.16 | -0.18 | -0.17 | -0.04 |
| Needle Cooling (NC) | -0.07 | 0.02 | 1.00 | -0.05 | -0.03 | -0.05 | 0.09 | 0.02 | -0.05 | 0.13 |
| Lead Dry Matter Content (LDMC) | -0.13 | -0.10 | -0.05 | 1.00 | 0.42 | 0.04 | 0.20 | 0.03 | 0.08 | 0.13 |
| Leaf Mass per Area (LMA) | -0.12 | 0.01 | -0.03 | 0.42 | 1.00 | 0.09 | 0.30 | 0.03 | 0.26 | 0.03 |
| Water User Efficiency (WUE) | -0.11 | -0.05 | -0.05 | 0.04 | 0.09 | 1.00 | -0.12 | 0.01 | 0.01 | -0.12 |
| Carbon to Nitrogen ratio (CN) | -0.36 | -0.16 | 0.09 | 0.20 | 0.30 | -0.12 | 1.00 | 0.23 | 0.09 | 0.14 |
| Huber Value (HV) | -0.12 | -0.18 | 0.02 | 0.03 | 0.03 | 0.01 | 0.23 | 1.00 | 0.01 | 0.16 |
| Stomatal Density (SD) | -0.09 | -0.17 | -0.05 | 0.08 | 0.26 | 0.01 | 0.09 | 0.01 | 1.00 | 0.03 |
| Twig Specific Density (TSD) | -0.06 | -0.04 | 0.13 | 0.13 | 0.03 | -0.12 | 0.14 | 0.16 | 0.03 | 1.00 |

Figure S1. Geographical location of the three provenance trial (grey triangles) and the seven provenance (grey circles). The black spruce range as define by Little (1971) is outlined in black, and the colored background represents the average annual climate moisture index (CMI) between 1981-2010. NAD83(CSRS) projection from statistics Canada was used (“NAD83(CSRS) Updates” 2024).

**Panel B**

**Panel A**

Figure S2. PCA of trait values. Panel A: Analysis on individuals from all sites. Panel B: Analyses on individuals at each site.

Figure S3. Selection gradients across sites for the trait and trait combinations under significant and marginally significant selection at Petawawa. P-values for t-tests are only shown for clinal selection gradients with climate.

a

b

c

a

b

c

a

b

c

a

b

c

a

b

c

a

b

c

a

a

b

a

a

b

a

a

b

a

b

c

Figure S4. Comparative reaction norms of provenances across sites for all the study traits. Black crosses show site means. For a given trait, sites with different letters indicate significantly different means (p < 0.05). See Table 1 for units and table S1 for provenance information.

## Supplementary Methods

### Trait measurements

Relationship between traits, physiological function and warmer, drier climates

To characterize investment in structural support at the needle and branch levels, leaf dry matter content (LDMC; g/g) and twig specific density (TSD; g/cm^3^) were measured. LDMC, the ratio of dry to water-saturated mass, reflects needle resource investment into structural support versus metabolic processes (Shipley *et al.* 2006) and has been shown to increase with drought (Anderegg *et al.* 2021). TSD affects mechanical support, storage and hydraulics (Pratt and Jacobsen 2017) and closely related traits such as wood density can increase with water limitation (Anderegg *et al.* 2021). Leaf mass per area (LMA, g/cm^2^) reflects the carbon cost of light interception and in global interspecific comparisons is an important indicator of plant carbon-use strategies (Westoby *et al.* 2002; Wright *et al.* 2004), with high values indicating high structural support and needle persistence, and low values indicating high photosynthetic rate. Among species, high LMA values has also been shown to correlate with tolerance to heat stress (Münchinger *et al.* 2023) and drought stress (Poorter *et al.* 2009; Scoffoni *et al.* 2014; Rosas *et al.* 2019). Carbon to Nitrogen ratio (CN; g g^-1^) reflects the nutrient concentration in the leaves. High nitrogen concentration (low CN) is largely driven by high concentration of RUBISCO and is therefore correlated with high photosynthetic rate (Reich *et al.* 1999). Water use efficiency (WUE; ‰) was measured to characterize the grams of water lost per unit of carbon fixed. We used carbon isotope ratio (δ^13^C) to estimate intrinsic water use efficiency (Ma *et al.* 2023). During photosynthesis, ^12^CO_2_ is preferentially fixed over ^13^CO_2_ primarily because the enzyme RUBISCO discriminates against ^13^CO_2_ and only binds it when ^12^CO_2_ is limited, which occurs when stomata are closed (Farquhar *et al.* 1982; Lambers *et al.* 2008). The ratio of ^13^C to ^12^C present in plant tissue, therefore reflects the amount of time leaves spent with their stomata closed. δ^13^C gives the ^13^C isotope composition relative to the primary reference scale of Vienna Pee Dee Belemnite. Within species, δ^13^C often increases with water limitation (Limousin *et al.* 2015; Hajek *et al.* 2016; Rosas *et al.* 2019; Ahrens *et al.* 2020; Csilléry *et al.* 2020; Anderegg *et al.* 2021; Rabarijaona *et al.* 2022; Lochin *et al.* 2024). Stomatal density (SD; number/cm) affects leaf gas exchange and is thus a strong correlate of both transpiration rate and photosynthetic rate (Lambers *et al.* 2008) and has been shown to vary within species with drought (Aragón *et al.* 2023). Huber value (HV; mm/g), the ratio of sapwood area to needle weight, characterizes water supply relative to water demand at the branch level (Carter and White 2009). High Huber values typically indicate increased conservative water use and thus increased drought avoidance (Mencuccini and Grace 1995; Mencuccini *et al.* 2019). It has been shown to increase within species with increasing water limitation (Rosas *et al.* 2019; Anderegg *et al.* 2021). To assess thermoregulation, needle cooling (NC; ºC) was measured as the difference between needle temperature and ambient temperature. Leaf cooling allows a leaf to partly control its temperature and stay within the optimal temperature range for photosynthesis (Michaletz *et al.* 2015). The readings from the thermal image were calibrated against the readings from a custom-built electronic thermometer with a TMP117 sensor with an accuracy of ± 0.1°C. The sensor plate of the electronic thermometer was placed inside each thermal image and the true temperature was manually recorded when each thermal picture was taken. Any difference between the true temperature and the temperature reading on the sensor plate from the thermal camera was used to correct the temperature readings of the needles within each thermal image. Chlorophyll concentration (CHL; mg/m^2^) was measured because it is strongly correlated with photosynthetic capacity (Lambers *et al.* 2008). While we do not expect photosynthesis to be directly selected for under warmer and drier climates, we expect it will be indirectly under selection for lower rates because photosynthesis comes at the cost of water loss, which we expect to be minimized. Needle length (NL; mm) affects total light capture and water loss of the plant, as well as needle thermoregulation by modifying the thickness of boundary layer and thus passive heat loss (Lambers *et al.* 2008).

On each branch, we measured traits on one- to four-year-old needles. This age range was selected because many needles that formed during the year of collection (age 0) were not yet mature and needles older than 4 years were frequently absent. Three traits were measured at the branch level (HV, WUE and CN, Table 1). The other seven traits were first measured from each needle age class, then averaged at the branch level by abundance-weighting by the relative needle biomass of each age class. The relative dry needle biomass was used for needle traits and relative dry branch biomass for TSD.

### References

**Ahrens CW, Andrew ME, Mazanec RA, *et al.*** **2020**. Plant functional traits differ in adaptability and are predicted to be differentially affected by climate change. *Ecology and Evolution* **10**: 232–248.

**Anderegg LDL, Loy X, Markham IP, *et al.*** **2021**. Aridity drives coordinated trait shifts but not decreased trait variance across the geographic range of eight Australian trees. *New Phytologist* **229**: 1375–1387.

**Aragón L, Messier J, Atuesta-Escobar N, Lasso E**. **2023**. Tropical shrubs living in an extreme environment show convergent ecological strategies but divergent ecophysiological strategies. *Annals of Botany* **131**: 491–502.

**Carter JL, White DA**. **2009**. Plasticity in the Huber value contributes to homeostasis in leaf water relations of a mallee Eucalypt with variation to groundwater depth. *Tree Physiology* **29**: 1407–1418.

**Csilléry K, Buchmann N, Fady B**. **2020**. Adaptation to drought is coupled with slow growth, but independent from phenology in marginal silver fir (*Abies alba* Mill.) populations. *Evolutionary Applications* **13**: 2357–2376.

**Farquhar G, O’Leary M, Berry J**. **1982**. On the relationship between carbon isotope discrimination and the intercellular carbon dioxide concentration in leaves. *Functional Plant Biology* **9**: 121.

**Girardin MP, Isabel N, Guo XJ, Lamothe M, Duchesne I, Lenz P**. **2021**. Annual aboveground carbon uptake enhancements from assisted gene flow in boreal black spruce forests are not long-lasting. *Nature Communications* **12**: 1169.

**Hajek P, Kurjak D, Von Wühlisch G, Delzon S, Schuldt B**. **2016**. Intraspecific variation in wood anatomical, hydraulic, and foliar traits in ten European beech provenances differing in growth yield. *Frontiers in Plant Science* **7**.

**Lambers H, Chapin FS, Pons TL**. **2008**. *Plant physiological ecology*. New York, NY: Springer New York.

**Limousin J, Yepez EA, McDowell NG, Pockman WT**. **2015**. Convergence in resource use efficiency across trees with differing hydraulic strategies in response to ecosystem precipitation manipulation. *Functional Ecology* **29**: 1125–1136.

**Little EL**. **1971**. *Atlas of United States trees. Volume 1. Conifers and important hardwoods.* Washington, D.C.: U.S. Dept. of Agriculture, Forest Service.

**Lochin P, Malherbe P, Marteau B, *et al.*** **2024**. The ant and the grasshopper: Contrasting responses and behaviors to water stress of riparian trees along a hydroclimatic gradient. *Science of The Total Environment* **952**: 175916.

**McKenney DW, Hutchinson MF, Papadopol P, *et al.*** **2011**. Customized Spatial Climate Models for North America. *Bulletin of the American Meteorological Society* **92**: 1611–1622.

**Mencuccini M, Grace J**. **1995**. Climate influences the leaf area/sapwood area ratio in Scots pine. *Tree Physiology* **15**: 1–10.

**Mencuccini M, Rosas T, Rowland L, *et al.*** **2019**. Leaf economics and plant hydraulics drive leaf : wood area ratios. *New Phytologist* **224**: 1544–1556.

**Michaletz ST, Weiser MD, Zhou J, Kaspari M, Helliker BR, Enquist BJ**. **2015**. Plant thermoregulation: energetics, trait–environment interactions, and carbon economics. *Trends in Ecology & Evolution* **30**: 714–724.

**Münchinger IK, Hajek P, Akdogan B, Caicoya AT, Kunert N**. **2023**. Leaf thermal tolerance and sensitivity of temperate tree species are correlated with leaf physiological and functional drought resistance traits. *Journal of Forestry Research* **34**: 63–76.

**NAD83(CSRS) Updates**. **2024**. https://webapp.csrs-scrs.nrcan-rncan.gc.ca/geod/tools-outils/nad83-docs.php. 20 May 2025.

**Poorter H, Niinemets Ü, Poorter L, Wright IJ, Villar R**. **2009**. Causes and consequences of variation in leaf mass per area (LMA): a meta‐analysis. *New Phytologist* **182**: 565–588.

**Pratt RB, Jacobsen AL**. **2017**. Conflicting demands on angiosperm xylem: Tradeoffs among storage, transport and biomechanics. *Plant, Cell & Environment* **40**: 897–913.

**Rabarijaona A, Ponton S, Bert D, *et al.*** **2022**. Provenance differences in water-use efficiency among sessile oak populations grown in a mesic common garden. *Frontiers in Forests and Global Change* **5**: 914199.

**Rosas T, Mencuccini M, Barba J, Cochard H, Saura‐Mas S, Martínez‐Vilalta J**. **2019**. Adjustments and coordination of hydraulic, leaf and stem traits along a water availability gradient. *New Phytologist* **223**: 632–646.

**Scoffoni C, Vuong C, Diep S, Cochard H, Sack L**. **2014**. Leaf shrinkage with dehydration: coordination with hydraulic vulnerability and drought tolerance. *Plant Physiology* **164**: 1772–1788.

**Shipley B, Lechowicz MJ, Wright I, Reich PB**. **2006**. Fundamental trade-offs generating the worldwide leaf economics spectrum. *Ecology* **87**: 535–541.

**Westoby M, Falster DS, Moles AT, Vesk PA, Wright IJ**. **2002**. Plant ecological strategies: Some leading dimensions of variation between species. *Annual Review of Ecology and Systematics* **33**: 125–159.

**Wright IJ, Reich PB, Westoby M, *et al.*** **2004**. The worldwide leaf economics spectrum. *Nature* **428**: 821–827.
